# Factors Affecting Spontaneous Endocytosis and Survival of Probiotic Lactobacilli in Human Intestinal Epithelial Cells

**DOI:** 10.3390/microorganisms10061142

**Published:** 2022-05-31

**Authors:** Diana Aurora Ramirez-Sánchez, Noemi Navarro-Lleó, Christine Bäuerl, Samuel Campista-León, José María Coll-Marqués, Gaspar Pérez-Martínez

**Affiliations:** 1 Facultad de Biología, Universidad Autónoma de Sinaloa, Culiacán Rosales 80040, Mexico; diana-ramirez-sanchez@hotmail.com (D.A.R.-S.); calsa68@hotmail.com (S.C.-L.); 2 Department of Microbiology and Ecology, Faculty of Medicine, University of Valencia, 46010 Valencia, Spain; noemi.navarro@uv.es; 3 Laboratory of Lactic Acid Bacteria and Probiotics, Department of Biotechnology, Instituto de Agroquímica y Tecnología de Alimentos, Consejo Superior de Investigaciones Científicas (CSIC) Valencia, 46980 Paterna, Spain; cbauerl@iata.csic.es (C.B.); jcoll@iata.csic.es (J.M.C.-M.)

**Keywords:** *Lacticaseibacillus paracasei*, *Lacticaseibacillus rhamnosus*, internalization, mechanism of endocytosis, clathrin, caveolin, intestinal epithelial cells, intracellular survival, transcytosis

## Abstract

Mutualistic bacteria have different forms of interaction with the host. In contrast to the invasion of pathogenic bacteria, naturally occurring internalization of commensal bacteria has not been studied in depth. Three in vitro methods, gentamicin protection, flow cytometry and confocal laser scanning microscopy, have been implemented to accurately assess the internalization of two lactobacillus strains—*Lacticaseibacillus paracasei* BL23 and *Lacticaseibacillus rhamnosus* GG—in Caco-2 and T84 intestinal epithelial cells (IECs) under a variety of physiological conditions and with specific inhibitors. First and most interesting, internalization occurred at a variable rate that depends on the bacterial strain and IEC line, and the most efficient was BL23 internalization by T84 and, second, efficient internalization required active IEC proliferation, as it improved naturally at the early confluence stages and by stimulation with epidermal growth factor (EGF). IFN-γ is bound to innate immune responses and autolysis; this cytokine had a significant effect on internalization, as shown by flow cytometry, but increased internalization was not perceived in all conditions, possibly because it was also stimulating autolysis and, as a consequence, the viability of bacteria after uptake could be affected. Bacterial uptake required actin polymerization, as shown by cytochalasin D inhibition, and it was partially bound to clathrin and caveolin dependent endocytosis. It also showed partial inhibition by ML7 indicating the involvement of cholesterol lipid rafts and myosin light chain kinase (MLCK) activation, at least in the LGG uptake by Caco-2. Most interestingly, bacteria remained viable inside the IEC for as long as 72 h without damaging the epithelial cells, and paracellular transcytosis was observed. These results stressed the fact that internalization of commensal and mutualistic bacteria is a natural, nonpathogenic process that may be relevant in crosstalk processes between the intestinal populations and the host, and future studies could determine its connection to processes such as commensal tolerance, resilience of microbial populations or transorganic bacterial migration.

## 1. Introduction

In the human gastrointestinal tract, continuous and bidirectional interactions are taking place between the bacterial communities and the intestine at different levels, giving rise to a truly mutualistic relationship [1]. Until present, the interaction of mutualistic bacteria with the host has been associated to bacterial cell wall components, surface proteins or secreted proteins, short chain fatty acids and other metabolites that travel in the lumen and diffuse through the mucus layer [2,3], but spontaneous internalization of wild type commensal bacteria [4,5,6] and probiotics [7,8,9,10] has been reported, suggesting that it could be a part of the natural interaction between the intestinal microbiota and the host mucosae. Bacterial entry into intestinal epithelial cells (IEC) has been classically associated to pathogenicity processes, therefore accumulated knowledge on the interactions and invasion of pathogens may show important clues on the internalization of mutualistic and commensal bacteria. For instance, *Escherichia coli*, *Campylobacter jejuni*, *Salmonella typhimurium* and *Pseudomonas aeruginosa* require the expression of caveolin-1 (Cav-1) for invasion of different types of cells and tissues [11]. Additionally, two well-known entry mechanisms into non-professional phagocytes have been described for *Listeria monocytogenes* (the zipper mechanism), and *S. typhimurium* and *E. coli* (the trigger mechanism), with both leading to the reorganization of the actin cytoskeleton and clathrin-mediated endocytosis [12,13]. A different mechanism is used by enteropathogenic *E. coli* (EPEC), where a needle-like injection (Tir) leads to the remodeling of the actin cytoskeleton forming a typical pedestal that recruits clathrin and AP2 endocytic machinery [14]. In contrast, Gram-positive bacteria, such as *Staphylococcus aureus* or *Streptococcus pyogenes*, have developed surface structures that bind to various extracellular matrix proteins (EMPs), such as fibronectin-binding protein (Fbp), that provoke entry by inducing the phosphorylation of FAK (protein associated with focal contact) and the substrate cortactin (Src) [15]. Hence, clathrin and caveolin-mediated host cell endocytosis are most frequent during the early steps of pathogen invasion.

Some works have described the spontaneous endocytosis of commensal and beneficial bacteria and the process involved generally required initial binding steps. Some probiotic lactobacilli have pili-like structures [16,17,18,19] or specialized surface proteins (slpA) [18,19] that efficiently bind to epithelial extracellular matrix proteins. Fibronectin-binding protein (FnbA) from *Weissella cibaria* inhibited *S*. *aureus* adhesion to mammary epithelial cells [20], and in fact different reports indicate that *Lactobacillaceae* can compete and inhibit the internalization of pathogens [8,9,21,22]. Other studies reported spontaneous, internalization of lactobacilli by epithelial cells [8,9,23], and genetic engineering works focused on the expression of surface pathogens’ adhesion proteins or single chain antibodies in *Lactococcus lactis*, *Lactiplantibacillus plantarum* and *Lacticaseibacillus paracasei*, to promote attachment and internalization by IEC in vitro and in vivo [8,24,25,26,27,28], hence achieving efficient bacto-transfection [8,24,25,26,27,28]. Nevertheless, the steps following adhesion, optimal epithelial cells status and the mechanisms required for lactobacillus internalization are as yet unknown. In contrast, the internalization of non-invasive *E.coli* has been well documented and it was bound to caveolin-mediated endocytosis and myosin light chain (MLC) phosphorylation in the terminal network (TW) region leading to brush border fanning (BB) in enterocytes, and this process was stimulated by IFN-γ [4]. Possibly, as occurred with pathogens and commensal *E. coli*, the internalization by the IEC of beneficial bacteria would require an inflammatory state and could also occur through endocytic pathways associated with lipid rafts and caveolin-1 [4,5].

*Lacticaseibacillus rhamnosus* and *L. paracasei* are very closely related species, yet with differential features. *L. rhamnosus* is a probiotic species widely studied in clinical trials for the treatment and/or prevention of various health conditions such as, ulcerative colitis [29], diarrhea [30,31] and atopic dermatitis [30]. There is also scientific evidence on the beneficial effect of *L. paracasei* in the reduction of antibiotic-associated diarrhea [32], attenuation of colitis [33] or in the prevention of infections caused by *Clostridium difficile* [34]. *L. rhamnosus* inhibits the internalization of enterohemorrhagic *E. coli* (EHEC) [35] and elegant works demonstrated the role of pili proteins from *L. rhamnosus* in binding to the host cells surface and phagocytosis [36,37,38]. Pioneer studies on the internalization of *L. paracasei* described the requirement of active sortases for competent bacterial internalization, hence the relevance of the LPXTG cell wall bound proteins in the internalization of this probiotic strain [9]. Nevertheless, the steps following specific binding and internalization of these two very relevant lactobacilli, and the mechanisms involved, are still unknown.

In this context, this work focused on the intimate interaction of lacticaseibacillus bacteria with host cells trying to determine physiological factors affecting IEC that may favor it and the possible endocytic process, from the initial steps to the eventual fate of probiotic bacteria in epithelial cells’ cytoplasm. Then, different assays and strategies have been developed to elucidate the possible mechanisms of endocytosis of two probiotic strains, *L. paracasei* BL23 and *L. rhamnosus* GG, by Caco-2 and T84 IEC, including the use of growth promoters, cytokines and specific inhibitors. Commonly available methods have been implemented to quantify and observe these processes like gentamicin protection assays, flow cytometry and epifluorescence and confocal laser scanning microscopy.

## 2. Materials and Methods

### 2.1. Bacterial Strains Growth Conditions

Bacterial strains *L. paracasei* BL23 (BL23) and *L. rhamnosus* GG (LGG) were regularly grown on MRS medium (BD™ Difco™ Lactobacilli MRS Broth; Thermo Fisher Scientific, Waltham, WA, USA) under static conditions at 37 °C for 24 h. For counting isolated colonies on petri dishes, MRS with 1.5% agar was used. The strain *L. paracasei* BL23 [pT1GRPC37] was expressing the red fluorescent (RFP) and green fluorescent (GFP) proteins [39], and it was used in the fluorescence/confocal microscopy and flow cytometry assays, as appropriate. This strain required selective pressure of 5 µg/mL of erythromycin (Erm^5^) in MRS and then it was grown under the same conditions. For the internalization assays described below, BL23 [pT1GRPC37], BL23 and LGG were inoculated in 10 mL MRS (with Erm5 for *L. paracasei* [pT1GRPC37]), and incubated at 37 °C for 24 h. Optical density of the cultures was measured at 550 nm (OD550) and, according to the calculated growth curve of these strains, the number of bacteria present (CFU/mL) was determined. The culture was centrifuged at 4000 rpm for 10 min, washed with sterile PBS and the pellet was diluted in medium supplemented for Caco-2 (ATCC HTB-37) or T84 (ATCC CCL-248) without antibiotics, with the appropriate dilution to obtain a multiplicity of infection (MOI) of 10^3^ bacteria/cell.

### 2.2. Epithelial Cell Culture and Other Reagents Used

IEC cultures of Caco-2 were maintained in DMEM (High Glucose, -Na-Pyruvate, Cat. No. L0102, Biowest, Riverside, MO, USA) supplemented with 10% heat inactivated fetal bovine serum (FBS), 1% non-essential amino acids (Cat. No. X0557, Biowest), 1% sodium pyruvate (NPY-B, Capricorn Scientific GmbH, Ebsdorfergrund, Germany), 1% L-glutamine (Cat. No. X0550, Biowest), 1% HEPES Buffer Solution (Cat. No. L0180, Biowest) and 1% penicillin-streptomycin (Cat. No. L0018-100, Biowest). The culture medium for the maintenance of the T84 cell line consisted of DMEM F-12 medium (Cat. No. L0090, Biowest), supplemented with 10% FBS, 1% penicillin-streptomycin, 1% HEPES buffer and 1% L-glutamine. For transcytosis studies, cells were seeded in cell culture polycarbonate Transwells inserts (Costar^®^; Cole-Parmer, Vernon Hills, IL, USA) (24 mm diameter; 3.0 μm pore size) at 2 × 10^5^ cells/mL for the Caco-2 and 6 × 10^5^ cells/mL for the T84. For the transcytosis assays, Lucifer Yellow (LY) (Sigma-Aldrich, St. Louis, MO, USA) was prepared at 50 µmol/L and propidium iodide (Sigma-Aldrich) at 1 mg/mL in PBS.

### 2.3. Standard Gentamicin Protection Assay and Reagents Used in Internalization Assays

This assay was used to estimate the amount of internalized bacteria in the epithelial cells and it was based on previous works by others with modifications [7,24,26]. Briefly, 24 well plates were seeded with 8 × 10^4^ cells/mL per well of Caco-2 and 2.6 × 10^5^ cells/mL per well of T84. Then, the plates were incubated at 37 °C with 5% CO_2_ until the Caco-2 reached 95–100% and the T84 reached 70–80% confluence. Before the co-incubation with bacteria, the culture medium was replaced by suitable fresh medium without antibiotics and the IECs were incubated for 2 h with BL23 and LGG with a MOI 1000 at 37 °C and 5% CO_2_. Four wells were randomly assigned in each plate for zero bacteria controls. Then, the cells were washed with PBS pH 7.4 and incubated with 500 μL of gentamicin (300 μg/mL) in IEC culture medium without antibiotics for 2 h, to eliminate non-internalized bacteria. Then, the cells were washed with PBS and lysed in 1 mL of cold 0.2% Triton X-100 and suitable dilutions were inoculated on MRS agar plates and incubated at 37 °C for 48 h. In order to study potential conditions affecting endocytosis, different chemokines, stimulants or inhibitors of endocytosis were added to the IEC cultures. The reagents used were interferon-γ (IFN-γ) (Cat No. 11343536, ImmunoTools, Friesoythe, Germany), cytochalasin D (Cat No. PHZ1063, ThermoFisher Scientific, Waltham, MA, USA), 5-(N-Ethyl-N-isopropyl) α-amiloride (EIPA) (Cat No. A3085, Sigma-Aldrich), rapamycin (Cat No. R0395, Sigma-Aldrich), dynasore hydrate (Cat No. 1202867-00-2, Sigma-Aldrich), ML7 (Cat No. I2764, Sigma-Aldrich) and epidermal growth factor (EGF) (Cat No. 11343407, ImmunoTools). They were dissolved in culture medium without antibiotics that was used to incubate the IEC cultures before the co-incubation with each bacteria. INF-γ was incubated for 48 h before the addition of bacteria to a final concentration of 1000 IU/mL or 100 IU/mL. For the treatments with other drugs the IECs were pre-incubated with those compounds for 1 h before the exposure to bacteria. These treatments included cytochalasin D (1 and 5 μg/mL), EIPA (50 μM and 25 μM), Rapamycin (10 and 20 ng/mL), EGF (50 and 100 ng/mL), *L. paracasei* BL23 derived proteins P40 (0.25 nM/mL) and P75 (0.025 nM/mL) [40], ML7 (10, 20 μmol/L) and dynasore (80 μmol/L). In the case of reagents prepared in DMSO (cytochalasin D, dynasore, EIPA, ML7 and Rapamycin), the final concentration of DMSO in the culture medium was not greater than 0.1%, and an appropriate control of cells incubated with 0.1% DMSO in the culture medium was included.

### 2.4. Analysis of Bacterial Endocytosis by Fluorescence and Confocal Microscopy

For this assay, Caco-2 or T84 cells were seeded on glass coverslips in 24-well plates and the procedure followed was similar to that described above. After pre-incubation with the stimulants or inhibitors (see above), the strain BL23 [pT1GRPC37] expressing RFP y GFP was added at a MOI 1000 and incubated for 2 h at 37 °C and 5% CO_2_. IECs were washed twice with PBS pH 7.4 and incubated for 2 h with gentamicin (300 μg/mL). After incubation, the cells were washed with PBS pH 7.4 and finally, fixed with 200 μL of paraformaldehyde (PFA) 4% for 10 min. Additionally, a time-lapse assay was carried out where bacteria were removed after 2 h, and then cells were incubated in the appropriate culture medium with gentamicin (300 μg/mL) during longer times (2, 4, 6, 8, 12, 24, 48 and 72 h).

Fixation was carried out with 200 µL of 4% PFA, as mentioned above, with three washes with 1 mL of PBS pH 7.4. In general for visualization, cells were stained with DAPI (4′,6-diamino-2-phenylindole; cat. D1306, ThermoFisher Scientific) and phalloidin-TRITC (phalloidin-tetramethylrhodamine B isothiocyanate; Cat No. P1951; Sigma-Aldrich). When required, instead of phalloidin-TRITC, a 1:400 mixture of WGA 594 (Wheat Germ Agglutinin Alexa Fluor 594; Cat no. W11262; ThermoFisher Scientific) was added and incubated for 15 min in the dark, and washed twice with 1 mL of PBS pH 7.4. Samples were mounted on slides with ProLong ™ Diamond Antifade Mountant (Cat. No. P36965, ThermoFisher Scientific) and the images were visualized and captured in the Nikon Eclipse E90i Fluorescence Microscope (Nikon Corporation, Tokyo, Japan) equipped with a 5-megapixels cooled digital color camera (DS-5Mc; Nikon corporation, Japan) using epifluorescence (using a mercury lamp, and the following Nikon filter blocks: for blue, UV-2E/C 340–380/435–485 or UV-2A 330–380/LP 420; for green, B-2E/C 465–495/515–555; for red, G-2a 515–565/565–615). To study epithelial cell viability by epifluorescence, the coverslips were incubated with 5 µg/mL of propidium iodide for 5 min before cell fixation in the dark. They were then washed with PBS and fixed with 4% PFA, washed again with PBS and 5 µg/mL of DAPI was added. The coverslips were then mounted in ProLong Gold mounting medium and allowed to dry in the dark at 4 °C overnight. Individual images or stacks were captured by the Nis Elements BR 3.2 software (Nikon Corporation, Japan) and some image processing operations were executed with Fiji software (ImageJ 1.49q Software, National Institutes of Health, Bethesda, MD, USA), such as color merge, stack-Z maximum projection and occasionally a subtract background of blue channel followed by a fully conservative enhanced contrast to optimize histogram distribution without pixel saturation. The Nikon objectives used were CFI Plan Fluor DIC M/N2 20X MRH00200 N.A. 0.5 Dry-Air; CFI Plan Fluor DIC M/N240X MRH00401 N.A. 0.75 Dry-Air; and CFI Plan Fluor DIC H/N2 100X Oil MRH01900 N.A. 1.3 Oil. The main epifluorescence image properties were: RGB 24 bits, a frame size of 1280 × 960 pixels, and the image dimensions of field of view were 85 × 65 μm for 100× objective (0.06 μm/pixel), 215 × 160 μm for 40× objective (0.1 μm/pixel), and 430 × 320 μm for 20× objective (0.34 μm/pixel).

For the confocal observations, the images were visualized in the Olympus FV1000 confocal laser scanning microscope mounted on a motorized inverted IX81 (Olympus Corporation, Tokyo, Japan) with a 60× immersion oil objective (Olympus UPLSAPO 60X O NA: 1.35) and equipped with a digital camera. The images were processed with the FV10-ASW 4.2 software (Olympus Corporation, Japan) employing the 405 nm laser (Blue) to detect DAPI, 488 nm laser (Green) to detect bacteria GFP and 559 nm laser for phalloidin-TRITC (Red; Alexa Fluor 568), using a distance of 0.5 μm for each field captured in Z. The main confocal image properties were: RGB 24 bits, an original frame size of 1024 × 1024 pixels, and the image dimensions of field of view were 212 × 212 μm for 60× objective (0.21 μm/pixel). Images shown in figures are a detailed zoomed selection of a maximum Z-projection of the XY frontal view, and XZ/YZ orthogonal views at the yellow lines.

### 2.5. Flow Cytometry Assays

Internalization of the strain *L. paracasei* BL23 [pT1-GR::p127] was analyzed by flow cytometry following the same conditions described above: MOI 10^3^ and 2 h of co-incubation with Caco-2 or T84 epithelial cells (under the same treatments mentioned above). The same assay was also performed with and without IFN-γ treatment at different times with Caco-2 (0, 30, 60, 120 min). After co-incubation, cells were washed twice with 1 mL of PBS pH 7.4 with 100 μL of trypsin-EDTA solution to detach and separate the epithelial cells, and then suspensions were incubated for 5 min at 37 °C. Finally, they were fixed with 300 μL of 4% paraformaldehyde (PFA) to have a final concentration of approx. 3%. Samples were stored at 4 °C in the dark until use. The samples were filtered with 50 μm Cuo Filcons Non Sterile (Cat no. 340632, BD BIOSCIENCES, San Jose, CA, USA). The fluorescent cell quantification was performed using the BD LSRFortesaTM cell analyzer flow cytometer (BD BIOSCIENCES), and the data processed using BD FACSDiva™ v6.2 software. For each interaction experiment, 50,000 events were analyzed.

### 2.6. Transcytosis, Transepithelial Electrical Resistance (TEER) and Paracellular Permeability

In order to determine whether BL23 and LGG had the ability to cross through the Caco-2 or T84 monolayer, transcytosis assays were performed as described before in [41,42,43], with slight modifications. Briefly, Caco-2 and T84 cells were seeded on transwell culture inserts (as explained above) and the plates were incubated for 10 and 17 days, respectively. During this period, the transepithelial electrical resistance (TEER) was determined with a specialized electrode (Millipore). The experiment started when reaching a minimum TEER value of 200 Ω.cm^2^ for the Caco-2 and 1000 Ω.cm^2^ for the T84 monolayers. To start the experiments, the culture medium was replaced by eukaryotic growth medium without antibiotics. Bacteria were inoculated at the apical compartment of the insert at a MOI of 10^3^, where LY (50 μmol/L) was added as control of the paracellular permeability. After 5 to 24 h of incubation (37 °C, 95% humidity, 5% CO_2_), the concentration of bacteria in the basolateral chamber was determined by inoculating serial dilutions on MRS-agar plates. The concentration of LY in the basolateral compartment was determined by measuring the fluorescence emission at 530 nm. When appropriate, the effect of the inhibitors ML-7 and dynasore was tested, preincubating the monolayer for 30 min before the addition of bacteria. IFN-γ was also tested, with 48 h preincubation in the basolateral compartment.

### 2.7. Analysis of L. paracasei [pT1-GR::p127] Colocalization with Clathrin and Caveolin Vesicles by Immunofluorescence

Caco-2 and T84 cells were grown on sterile 10–12 mm coverslips to approximately 80% confluence, then a bacterial suspension of *L. paracasei* [pT1-GR::p127] was added to a MOI 10^3^ and the incubations proceeded as indicated before. After incubation for 2 h, two washes with PBS were performed and eukaryotic growth medium containing 300 μg/mL gentamicin was added. After 2, 24 and 48 h, 3 washes with PBS were performed and the cells were fixed with 4% paraformaldehyde (PFA) at room temperature (RT) for 10 min. After performing 3 washes with PBS, the cells were permeabilized with 0.2% Triton X-100 for 10 min at RT, 3 washes were performed with PBS, and nonspecific binding blocking was performed for 1 h at RT with 4% bovine serum albumin (BSA) in PBS. All subsequent operations were carried out in the dark. The blocking solution was then removed and the cells were incubated with mouse monoclonal (anti-clathrin or anti-caveolin1) primary antibody (1:400) in blocking solution overnight at 4 °C. Six washes with PBS were performed to remove the unbound primary antibody and then AlexaFluor 594-conjugated anti-mouse secondary antibody (1:500) was added in a blocking solution, and incubated for one hour at room temperature. After six washes with PBS it was fixed again with 4% PFA for 10 min, washed three times with PBS and incubated with DAPI 5 µg/mL for 10 min at RT. Then, the coverslips were washed three times with PBS and mounted on a slide with ProLong Gold mounting medium and dried at 4 °C overnight. Images were acquired on the Nikon Eclipse E90i Fluorescence Microscope (Nikon Corporation, Japan) and processed with Nis Elements BR 3.2 software (Nikon Corporation, Japan).

### 2.8. Determination of Relative Intestinal Alkaline Phosphatase Activity

Alkaline phosphatase activity was determined by measuring the hydrolysis of p-Nitrophenyl phosphate (p-NPP) to inorganic phosphate and p-Nitrophenol (p-NP) a soluble, yellow end product, as previously described in [44]. The released p-NP was measured spectrophotometrically at 405 nm. The total protein was quantified using the “Bicinchoninic Acid Protein Assay Kit” (Sigma-Aldrich). In this way, intestinal alkaline phosphatase activity (nmol p-NP/min) relative to the amount of protein (mg) was calculated.

### 2.9. Statistical Analysis

All the experiments were performed with four replicates and three independent repetitions. The data were analyzed with the IBM SPSS Statistics version 23 and GraphPad Prism7 software through Mixed Variance Analysis, with two fixed factors (cell line, bacteria) and two random factors (day and well). The treatments were compared with the Bonferroni or Tamhane multiple comparison test, considering a *p* ≤ 0.05 as significant.

## 3. Results

### 3.1. Spontaneous Internalization of BL23 and LGG by Caco-2 and T84

**Figure 1:**
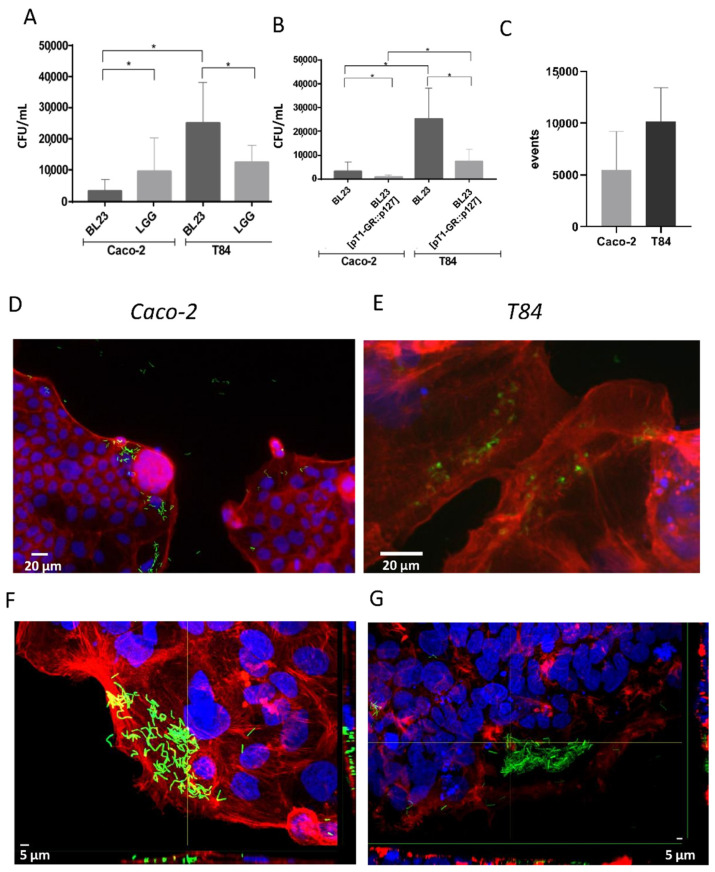
Different techniques used to determine spontaneous internalization of lactobacilli. (**A**) Internalization of BL23 and LGG in Caco-2 and T84 determined by the gentamicin protection method, and data represent means of CFU per mL specific assays and controls from other assays (a total of 64 experiments, with three replicates per assay, a total of 192 determinations), bars indicate standard deviations. (**B**) Assay showing differences in the internalization efficiency between BL23 and the recombinant strain carrying plasmid pT1-GR::p127 used in microscopic observations, as it allowed the expression of fluorescent proteins GFP and RFP (results from four separate assays, with three internal replicates per condition). (**C**) Internalization of BL23 [pT1-GR::p127] determined by flow cytometry in Caco-2 and T84; graph represents the means and standard deviation of red fluorescent events in two different assays with at least three determinations. Differences between groups compared with Tamhane’s test. * *p* < 0.001. (**D**,**E**) Epifluorescence photographs showing internalization of BL23 [pT1-GR::p127] by (**D**) Caco-2 and (**E**) T84 cells. White scale bars represent 20 µm. (**F**,**G**) confocal microscopy photographs showing internalization of BL 23 [pT1-GR::p127] by (**F**) Caco-2 and (**G**) T84 epithelial cells; orthogonal projections are shown at the bottom and right hand side of each photograph. White scale bars represent 5 µm.

Gentamicin protection assays were implemented in order to determine the spontaneous internalization of the probiotic strains BL23 and LGG by Caco-2 and T84 epithelial cell cultures. These assays showed that BL23 was internalized more efficiently in T84 cells than in Caco-2 (*p* < 0.001). In contrast, LGG behaved similarly in both cell lines, and as a consequence when comparing both lactobacilli, the BL23 uptake was lower than the LGG in Caco-2 but significantly greater than the LGG in the T84 (Figure 1A). This suggested that elements determining the internalization of BL23 and LGG could be different and that possibly the ligands required for BL23 uptake were more abundant in T84.

Other strategies using fluorescent probes were optimized to monitor the uptake of probiotic bacteria. For this purpose, a recombinant derivative of BL23 was used, BL23 [pT1-GR::p127], that expressed GFP and RFP. Despite that this recombinant strain showed a proportionally lower efficiency of internalization than the wild type, it was considered sufficient to follow internalization under the epifluorescence microscope (Figure 1B) and to monitor fluorescent events by flow cytometry (Figure 1C). Observation by epifluorescence microscopy showed that bacterial cells were not evenly distributed over the layer of epithelial cells, but bacteria were predominantly found associated to marginal regions of the epithelial cells’ monolayers in low confluence Caco-2 as well as in T84 (Figure 1D,E), a fact that would be investigated further (see below). In areas rich in bacteria, numerous whole, and apparently intact bacteria were observed inside the epithelial cells cytoplasm (Figure 1F,G), which certified the internalization determined by other methods.

### 3.2. Influence of the Physiological Status of Epithelial Cells on Internalization of BL23 and LGG

In order to determine if the physiological status of the epithelial cells had any effect on bacterial uptake, gentamicin protection assays were carried out after cells were stimulated with a pre-incubation with IFN-γ (100 and 1000 IU/mL) or EGF (50 and 100 ng/mL), as they regulate different cellular processes. IFN-γ is expressed and regulates inflammation and autolysis and alters tight junctions and the barrier function, while the epithelial growth factor (EGF) stimulates cell division.

#### 3.2.1. IFN-γ Treatment

**Figure 2:**
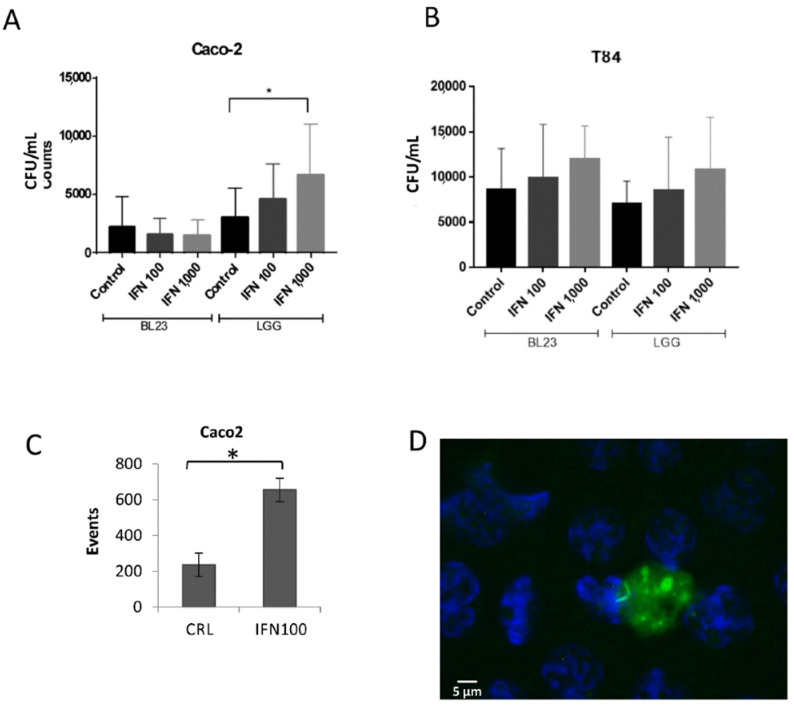
Gentamicin assays used to determine internalization of BL23 and LGG after the treatment of epithelial cells with 100 and 1000 UI/mL IFN-γ. (**A**) Internalization using Caco-2 and (**B**) T84 cells. Data shown are means of internalized bacteria per mL with standard deviation in all experiments (results from four separate assays, with three internal replicates per condition). (**C**) Effect of 100 UI/mL IFN-γ on the internalization of BL23 [pT1-GR-p127] determined by flow cytometry; graph represents the means and standard deviation of red fluorescent events in two different assays with at least three determinations. (**D**) Selected epifluorescence microscope image of Caco-2 cells incubated with GFP-expressing *L. paracasei* BL23 [pT1-GR::p127] after the treatment with 1000 U/mL IFN-γ. The white scale bar represents 5 µm. Differences between groups compared with Tamhane’s test. * *p* < 0.001.

Interestingly, stimulation with IFN-γ led to a higher internalization of LGG in the Caco-2, but it had no effect on BL23 uptake (Figure 2A), when determined as bacterial survival by gentamicin protection assays. In the T84, there was an apparent concentration dependent effect in both strains, but differences were not significant (Figure 2B). By flow cytometry a significantly higher proportion of fluorescent recombinant BL23 bacteria was associated to the INF-γ (100 IU/mL) treatment in Caco-2 (Figure 2C). Microscopic observation of the internalization after IFN-γ induction showed the presence of intracellular BL23 [pT1-GR::p127] bacterial cells, but also discrete diffuse stains of both fluorescent proteins were frequently encompassed in apparent vesicles that also contained bacterial cells in the process of degradation (Figure 2D). Microscopic observations may explain the discordance of quantitative data obtained by gentamicin protection and flow cytometry assays. In the first case, the data referred exclusively to colony forming units (CFU), that is viable units, while in the flow cytometry individual fluorescent events were quantified, which represented epithelial cells that may have engulfed one or more bacteria (Figure 1D–G), and where epithelial cells containing autolysis vesicles with non-viable bacteria would be counted as a fluorescent event.

#### 3.2.2. Active Growth Influences Internalization

**Figure 3:**
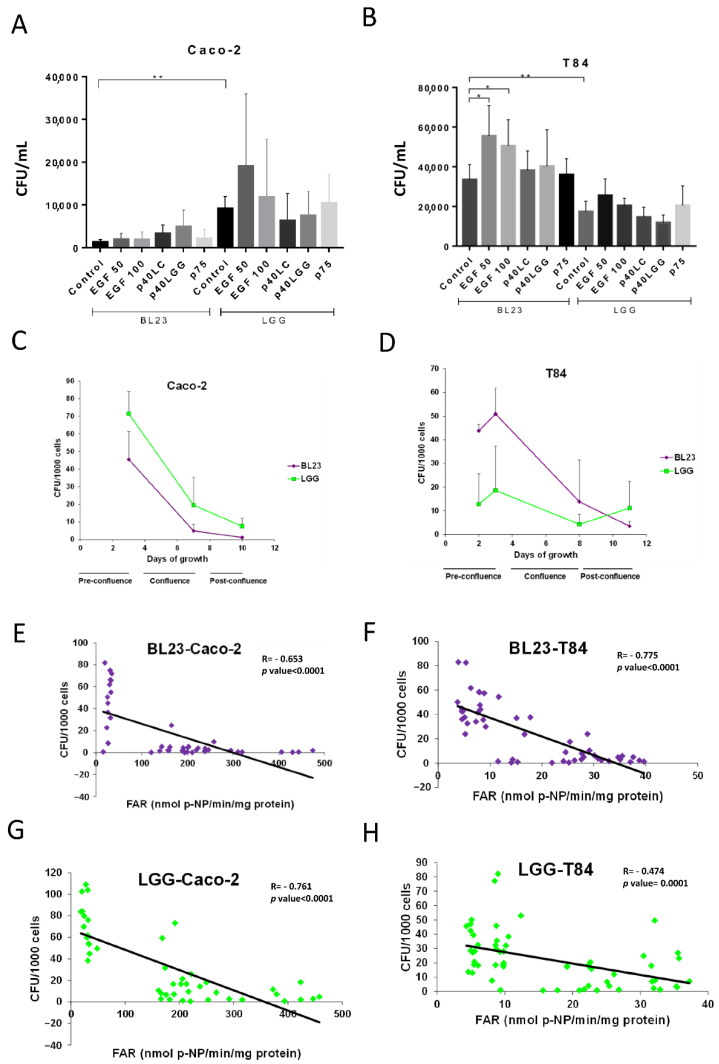
Relationship between growth stage and bacterial internalization. Graphs show gentamicin internalization assays after treatment with 50 and 100 ng/mL EGF on BL23 and LGG uptake by (**A**) Caco-2 and (**B**) T84 cells. The experiments were carried out with five replicates in triplicate and the differences between groups compared with the Bonferroni test (**A**,**B**) * *p* < 0.01; ** *p* < 0.001. Internalization was also determined at different confluence stages (pre-confluence, confluence, post-confluence) in the IEC of (**C**) Caco-2 and (**D**) T84. Internalization vs. differentiation of IEC—given as relative intestinal alkaline phosphatase activity (FAR)—was studied with BL23 (**E**) by Caco-2 and (**F**) by T84, and with LGG (**G**) by Caco-2 and (**H**) by T84. Pearson correlation analysis (**C**–**F**). Where R: Pearson’s correlation coefficient, *p*-value ≤ 0.0001.

Initial microscopic observations (Figure 1D–G) showed abundant bacterial forms internalized in the periphery of the cells confluence that were possibly actively growing cells. When the cell cultures were stimulated with EGF, the internalization of BL23 significantly increased in T84, while there was an apparent increase of LGG uptake in both cell lines, although it was not significant (Figure 3A,B). In contrast, no significant effect could be determined by flow cytometry (Supplementary Information, Figure S1A–D). In this assay, the purified proteins P40 and P75 from *L. paracasei* BL23 and P40 from LGG were also included, as they stimulate epidermal growth factor receptor (EGFR) phosphorylation and signaling [45]. A tendency to increase the internalization of BL23 by Caco-2 and T84, and a reduction of LGG uptake by the T84, was observed when stimulated with P40 from the BL23 and LGG, but with no significance. In order to study the effect of active growth, new assays were designed. A time-lapse assay was performed to determine internalization at different confluence stages (pre-confluence, confluence and post-confluence) for Caco-2 (day 3, 7 and 10) and T84 (day 2, 3, 8, 11). In both, the T84 and Caco-2 internalization of BL23 and LGG was much higher in the pre-confluent state and decreased significantly with time as the confluence advanced (*p*-value < 0.0001) (Figure 3C,D).

Both strains followed an identical pattern. As shown before, the internalization efficiency of LGG was significantly greater than the BL23 in Caco-2 (*p*-value = 0.001) (Figure 1A and Figure 3C). The LGG showed a constant uptake profile by the T84 (Figure 3D). In this cell line, the internalization frequency of the BL23 was much greater at an early growth stage and it decreased with confluence as occurred in the Caco-2 (Figure 3C). These data suggest that bacterial entry, especially BL23, depends on a state of active growth of the IECs. In order to determine if these internalization differences were also bound to the differentiation process of epithelial cells, relative FAR activity was determined [44]. Regression analysis showed that internalization had a significant inverse correlation with cell differentiation (Pearson’s correlation, *p*-value ≤ 0.0001) (Figure 3E–H). Despite that, these assays showed that undifferentiated, actively growing epithelial cells displayed optimal internalization. The following experiments used IEC at confluence with the aim of achieving conditions comparable to the previous assays.

### 3.3. Analysis of Likely Mechanisms of Internalization

#### 3.3.1. Effect of Inhibitors on Internalization

**Figure 4:**
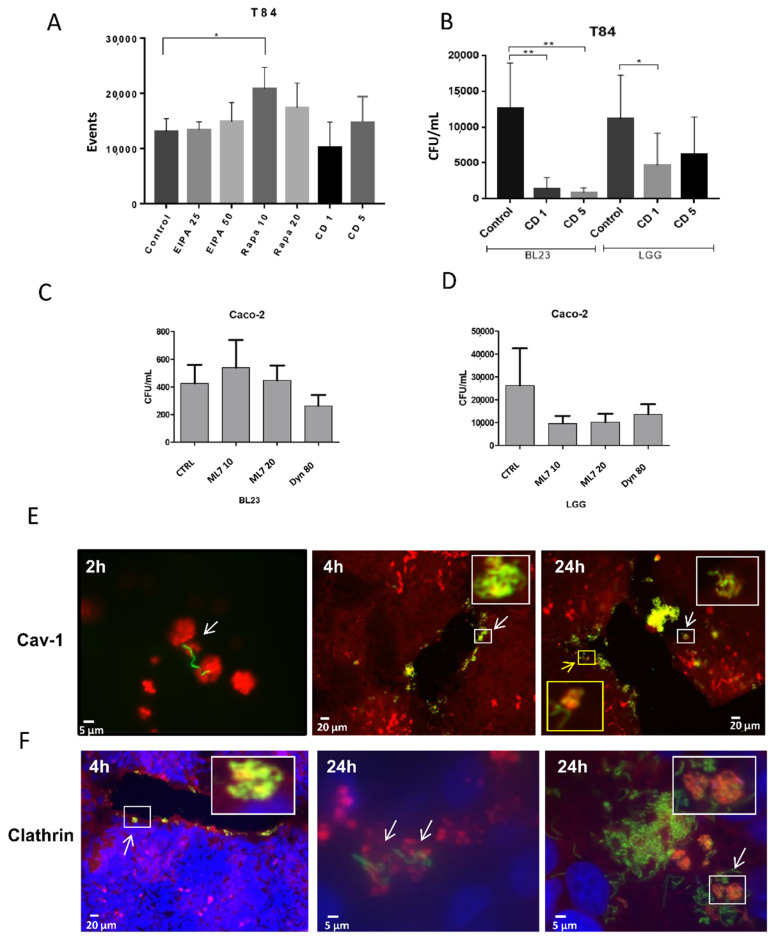
Bacterial uptake inhibition by different specific drugs and co-localization with clathrin and caveolin. Internalization was determined by (**A**) flow cytometry and (**B**–**D**) gentamicin protection assays. (**A**) Relative number of events found by flow cytometry after co-incubation of T84 with BL23 [pT1-GR::p127], treated with EIPA, rapamycin and cytochalasin D at the indicated concentrations. (**B**) Internalization assay of BL23 and LGG after gentamicin protection assay of T84 incubated with cytochalasin D. (**C**) Internalization of BL23 in Caco-2 cells treated with ML7 and dynasore at the indicated concentrations. (**D**) Internalization of LGG in Caco-2, same as (**C**). The experiments were performed with five replicates in triplicate and the differences between the groups compared with the Bonferroni and Tamhane tests, * *p* ≤ 0.05; ** *p* < 0.001. (**E**) Figures represent immune co-localization caveolin-1 with BL23 [pT1-GR::127] in cell line T84 at different times (2, 4 and 24 h). The blue channel revealing DAPI stained nuclei was deselected because the presence of blue fluorescence hampered the definition of caveolin-1 vesicles. The white scale bar represents 5 µm or 20 µm as indicated. (**F**) Figures represent immune co-localization clathrin with BL23 [pT1-GR::127] in cell line T84 at different times (4 and 24 h). In (**E**,**F**) bacteria are observed in green (GFP), conjugated anti-caveolin-1 and anti-clathrin are labelled in red (AlexaFluor594) and nuclei are observed in blue by DAPI. The white scale bar represents 5 µm or 20 µm as indicated.

Specific inhibitors such as 5-(N-(N-Ethyl-N-isopropyl) amiloride (EIPA), rapamycin, cytochalasin D, ML7 and dynasore were used in an attempt to identify the possible factors involved in the internalization of the probiotic lactobacilli BL23 and LGG. EIPA had no significant effect on the internalization of BL23 and LGG in both cell lines when determined by any of the methodologies used (Figure 4A and Supplementary information, Figure S2A–E). Rapamycin showed no apparent effect on the uptake of either bacteria in the IEC by the gentamicin protection assay (Supplementary information, Figure S2C,D), but a significant effect was found by flow cytometry in the uptake of BL23 [pT1-GR::p127] with low rapamycin concentrations in the T84 (Figure 4A). Since rapamycin induces autolysis, it may possibly lead to a reduction in the viable bacterial counts when determined by a gentamicin protection assay, a similar effect to that suggested for IFN-γ above.

Cytochalasin D treatment detached the monolayer of Caco-2 cells, hence only the T84 could be assayed by gentamicin protection. Both concentrations used of this inhibitor (1 and 5 µg/mL) significantly reduced BL23 and LGG internalization (Figure 4B). A reduction in events—but not significant—was detected by flow cytometry (Supplementary Information, Figure S3C,D). These data indicate that correct actin polymerization is required for the intracellular transition of lactobacilli. ML7 and dynasore are specific inhibitors of bacterial endocytosis. ML7 inhibits myosin light chain kinase (MLCK) and reduces the terminal web arc formation and brush border fanning, while dynasore blocks the GTPase activity of dynamin that is required for clathrin and caveolin dependent endocytosis (see details below). As occurred with the cytochalasin D, ML7 and dynasore were not water soluble, hence, equivalent amounts of DMSO were added to the controls. No effect was detected in BL23 and a partial reduction in LGG internalization was observed both with ML7 and dynasore, but without statistical significance (Figure 4C,D). This may be due to the LGG endocytosis being partially associated to clathrin- and/or caveolin-dependent endocytosis mechanisms, while BL23 possibly uses different mechanisms. In the T84 cell line, neither the BL23 nor LGG internalization appeared to be affected by any of the treatments (Supplementary Information, Figure S2E).

#### 3.3.2. Immunofluorescence Co-Localization of BL23 [pT1-GR::p127] with Clathrin and Caveolin-1

Anti-caveolin-1 and anti-clathrin antibodies were detected in Caco-2 and T84 cells previously incubated with BL23 [pT1-GR::p127] for different times (2–24 h). Both the caveolin-1 and clathrin co-localized with BL23, but of note, this phenomenon was only occasionally observed. Anti-caveolin antibodies revealed an accumulation of caveolin-1 in vesicular structures, that were rarely associated with bacteria at 2 h of incubation. After 4 h of incubation, some clusters of bacteria forming tangles close to the caveolin-1 vesicles could be observed at the periphery of the T84 monolayer, but, it was difficult to find an association of bacteria with caveolin after 24 h (Figure 4E). Associations were observed after short incubation times and not associated to bacterial degradation processes. This indicated that BL23 entry would partially depend on caveolin. The co-localization of BL23 with clathrin could only be seldom detected after short incubation times with bacteria; however, a higher number of clathrin-BL23 associations were observed on 24 h incubations (Figure 4F), which could be related to degradation processes after the bacterium had been internalized. Different types of clathrin vesicles containing and surrounding fluorescent BL23 bacteria could be observed (Figure 4F). This may suggest that entry of lactobacilli may not totally be associated with a clathrin-dependent mechanism, but also when proceeding to autolysis. Observation of BL23 [pT1-GR::p127] internalization by Caco-2 followed a very similar pattern as in the T84 (data not shown).

### 3.4. Consequences of Probiotic Internalization in the Interaction with Host Cells

#### 3.4.1. Intracellular Survival

**Figure 5:**
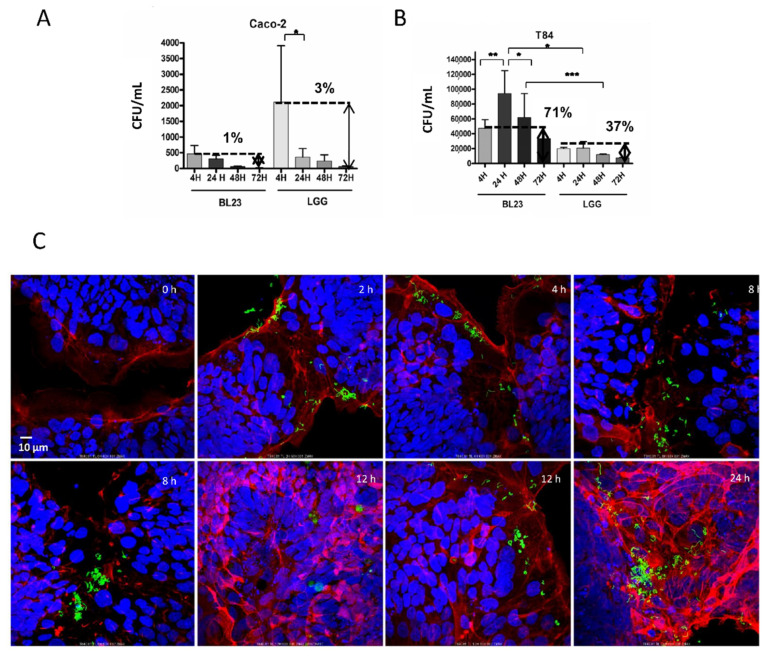
Survival of BL23 and LGG in the cytoplasm of T84 and Caco-2 at different times (4 h to 3 days). Gentamicin internalization. (**A**) Number of cfu/mL after incubation of BL23 and LGG with Caco-2 for the times indicated (4, 24, 48 and 72 h). (**B**) The same as in A, but in T84. (**C**) Confocal microscopy image of the internalization process of BL23 [pT1-GR::p127] by T84 after 2 h of co-incubation with bacteria and extended for 24 h with gentamicin. The experiments were performed with five replicates in triplicate and the differences between groups compared with the Bonferroni test. *p*-value ≤ 0.05 (*), *p*-value ≤ 0.001 (**), *p*-value ≤ 0.0001 (***). (**C**) Confocal microscopy image of the internalization process of BL23 [pT1-GR::p127] by T84 after 2 h of co-incubation with bacteria and extended for 22 h with gentamicin. Fluorophores legend: DAPI in blue, GFP in green, Actin in red. Objective used: 60×.

When internalized BL23 [pT1-GR::p127]—expressing GFP—were observed after one or more days, fluorescent, apparently viable bacterial cells persisted inside the epithelial cells and coexisted with autolysis vesicles (Figure 5C). Therefore, gentamicin protection assays were modified accordingly and, after bacterial addition, the time of incubation with gentamicin was extended for up to 72 h. After this time, gentamicin retained the antibacterial activity and the BL23 and LGG survived inside Caco-2 cells for 24 h, decreasing after three days (72 h) to 1% and 3%, respectively (Figure 5A). Surprisingly in the T84, the BL23 counts increased significantly after 24 and 48 h, suggesting that lactobacilli may duplicate in the IEC cytoplasm. Survival after 72 h was as much as 71% of the initial counts. The remaining intracellular LGG bacterial cells in the T84 after 1, 2 and 3 days were proportionally more than in Caco-2 (Figure 5B).

When the procedure was performed in T84 cells with non-viable cells of BL23 [pT1-GR::p127], treated with gentamicin (300 µg/mL) and chloramphenicol (25 µg/mL), no CFUs were scored, but under the confocal microscope, intracellular green fluorescent units could be detected with abnormal morphologies (Supplementary Information Figure S4). This suggests that non-viable bacteria are also endocyted.

#### 3.4.2. Biofilm Formation

Microscopic observations of fluorescent BL23 often showed an occasional accumulation of bacterial cells at the surface of the epithelial cells’ monolayer (Supplementary Information, Video S1, T84C81_60x.mp4). In other tissues, biofilm formation favors bacterial attachment to the cell surface. Hence, the ability of BL23 and LGG to generate biofilms was determined with crystal violet. There was a significant difference in biofilm formation between the BL23 and LGG, as LGG produced a significant amount of biofilm (Supplementary Information Figure S5). This may explain why the LGG was internalized with equal efficiency by the Caco-2 and T84.

#### 3.4.3. Cytotoxicity of Lactobacilli

In order to determine if internalized bacteria would affect the viability of IEC Caco-2 and T84, cells were incubated with BL23 [pT1-GR::p127] for 4, 24 and 48 h. Cell viability was evaluated under epifluorescence microscope by comparing viable nuclei stained with DAPI against propidium iodide stained nuclei (non-viable). Cell counts showed that there was no significant association between non-viable nuclei and fluorescent lactobacilli (Table S1).

#### 3.4.4. Transcytosis

Since probiotic lactobacilli can survive in the cytoplasm when grown on solid supports, there was a possibility that they could be transferred to the basolateral side of the monolayers with no effect on tissue integrity. Hence, transcytosis of BL23 and LGG in Caco-2 and T84 monolayers was assayed with transwell inserts. Monolayer integrity was monitored by TEER and Lucifer yellow. Under our experimental conditions, no CFUs were recorded after the incubation of T84 with either bacteria.

Caco-2 showed an excellent stability in the transwell inserts with time up to 24 h. Table 1 shows transcytosis counts where the endocytosis inhibitor dynasore was tested. In all cases, the TEER remained stable between 200 and 230 and the permeability of LY was never greater than 1.98%.

Counts in the basolateral compartment showed that transcytosis of BL23 across Caco-2 monolayer occurred at a higher rate than LGG, despite that the internalization rate of LGG was greater in this cell line (Figure 1A). The presence of dynasore had no apparent effect.

## 4. Discussion

Numerous research articles have studied gut microbiota interaction with the host, with most of them focusing on population dynamics and functional responses of the host and health related issues [46], but non-pathogenic constituents of the gut microbiota undertake very close interactions with the mucosal tissues, which include the uptake of commensal and beneficial bacteria by epithelial cells. The first objective of this work was studying the intimate interaction of probiotic lactobacilli with epithelial cells and finding conditions and factors that promote it, shedding some light on its mechanisms.

Here, different techniques were implemented to study internalization because it was necessary to cover as many aspects of the process as possible. The gentamicin protection assay is quite labor intensive and it is based on the estimation of viable bacteria (or rather CFU) protected from gentamicin by the cell membranes in an epithelial cell culture. Microscopic images clearly showed that several bacteria could be internalized by a single epithelial cell, but also some IECs were detected with fluorescent vesicles apparently containing damaged bacteria. As a consequence, each fluorescent event detected by flow cytometry might have represented one epithelial cell loaded with one or more bacteria, therefore underestimating the actual number of viable bacteria internalized. Alternatively, flow cytometry will also detect events that would correspond to cells containing fluorescent vesicles, but not necessarily viable bacteria, especially after treatments and conditions inducing autolysis.

This work unequivocally confirmed that BL23 and LGG are internalized by intestinal epithelial cells with a different efficiency that depends on the bacterial strain used. LGG showed the same entry efficiency in both cell lines but BL23 was taken up at very different rates by Caco-2 and T84. The highest rate was found in the BL23 internalization by T84. Microscopic observations revealed that cells at the periphery of the confluent monolayers were richer in bound fluorescent bacteria than the rest of the culture. In order to determine if active growth favored internalization, IEC were stimulated with EGF, observing a notable increase with gentamicin protection assays, possibly because it stimulated clathrin dependent endocytosis and macropinocytosis. Nonetheless, higher concentrations of EGF had a reduced effect as it may have increased lysosomal content degradation and an EGFR mediated increase of mechanistic target-of-rapamycin complex-1 (mTORC1) activity and autolysis [47,48]. Sensibly, growth and proliferation in cell cultures requires great nutrient input along with biochemical signals that initiate anabolism. A practical consequence here was a greater bacterial uptake by the IEC at the early growth phase of the cultures (pre-confluence) vs. confluent phase, both with the Caco-2 and T84 and with the two bacterial strains, BL23 and LGG. Additionally, internalization was inversely related to the differentiation state of the cells.

The two bacterial strains used in this study (BL23 and LGG), produce the secreted, biologically active proteins, P40 and P75, with an anti-apoptotic (pro-proliferative) effect linked to phosphorylation of the EGFR receptor and the Erk/Akt pathway [45,49]. Further, its anti-inflammatory effect and autolysis induction has been recently shown [50]. Those signal transduction pathways may be related to internalization rates, therefore, it seemed interesting to perform the same assay using the P40 and P75 proteins, but we did not observe a significant effect, with only a small decrease in LGG internalization (viable counts) when the cells were incubated with P40, the meaning of which will be further studied.

IFNγ-induction is bound to inflammatory processes [51], and it is involved in the lysis of pathogens [52], but IFNγ has also been reported to be related to *E. coli* endocytosis and transcytosis in T84 cultures [53]. In this work, a significant increase in the internalization of LGG was observed in Caco-2 cells stimulated with IFNγ and an increase in counts, but not significant, in T84. The differences observed by flow cytometry using BL23 and Caco-2 were very remarkable. Since the incubation conditions were identical, this difference may be due to the bacterial lysis induced by IFNγ, as observed by epifluorescence microscopy, which suggests that the inflammatory condition increased the internalization rate, but that it would also reduce intracellular survival due to bacterial lysis.

In order to define if the internalization of probiotic lactobacilli is bound to macropinnocytosis or other forms of endocytosis, different specific inhibitors were used. For example, 5-(N-Ethyl-N-isopropyl) α-amiloride (EIPA) is supposed to inhibit macropinocytosis by blocking proton pumps, hence affecting actin remodeling by GTPases (46), but no significant effect was detected. Cytochalasin D blocks actin polymerization [54] directly affecting endocytic processes at the apical surface of EIC [55], but in contrast to EIPA, cytochalasin D produced a drastic reduction in BL23 and LGG internalization in the T84 cells, indicating that the entry of these lactobacilli into eukaryotic cells requires well-structured actin microfibers and/or endocytic processes that rely on actin microfilaments. ML7 is a myosin light-chain kinase (MLCK) inhibitor acting as an endocytosis inhibitor [56], and dynasore acts on dynamin, a GTPase required for the fission of membranes during clathrin mediated endocytosis [57]. No significant effect was detected for either of these inhibitors, although we observed a tendency to some reduction in LGG uptake by Caco-2. This suggests a different mechanism to that described for non-invasive *E. coli* in a mouse model [4], and makes it difficult to explain probiotic internalization through canonical endocytosis. The participation of endocytosis mediated by clathrin and caveolin on the entry of lactobacilli into eukaryotic cells was also tested by histochemical methods. Microscopic observations indicated that sometimes both clathrin and caveolin-1 vesicles colocalized with fluorescent BL23 bacteria, but bacteria were frequently not colocalized with these proteins, suggesting that perhaps LGG and BL23 are internalized by endocytic processes that do not require clathrin nor caveolin-1 [54]. This underlined the differences between the uptake mechanisms of probiotics and pathogenic Gram-negatives, as the latter are endocyted through dynamin, clathrin-mediated processes [58].

Rapamycin is the canonical inhibitor of mTOR, an effector that stimulates anabolic processes, macropinocytosis and it inhibits catabolic processes such as autophagy [48]. Interestingly, a significant increase in internalization was observed by flow cytometry in T84 incubated with rapamycin, but no increase in surviving bacteria (CFU) was detected after gentamicin protection assays. This suggested that rapamycin may be inducing internalization, simultaneously activating autolysis.

In this work we have also explored the consequences of the internalization of bacteria for the epithelial tissue. Surprisingly, we were able to verify that, both BL23 and LGG survive for long periods within epithelial cells without affecting the cell viability and the level of survival inside Caco-2 cells was lower than in T84, particularly in the case of BL23.

These bacteria survive inside T84 cells for up to 72 h, even with an increase in viable cells at 24 h, which could be interpreted as a certain bacterial proliferation in the cytoplasm of these epithelial cells. Further, if a similar situation would occur in vivo then it would allow the bacteria to survive under adverse conditions and return to the intestinal lumen to repopulate the microbiota. Transcytosis is a phenomenon that has already been demonstrated in non-pathogenic commensals and which may also be occurring with probiotic strains, for which future in vivo studies will show the consequences of transcytosis for the organism. In this case, probiotic bacteria would pass to the lower layers of the mucosa, and they may migrate through the lymphatic or blood vessels to other organs.

Consistent behavior of *L. rhamnosus* might be related to an equally efficient interaction with the cell surface in Caco-2 and T84 or the biofilm formation, both mediated by pili proteins that have been proven to participate in this process [37].

## 5. Conclusions

Spontaneous internalization of *Lacticaseibacillus paracasei* BL23 and *Lacticaseibacillus rhamnosus* LGG probiotic bacteria by IEC cultures takes place predominantly in actively growing cells. It is induced by growth factors and inflammatory conditions, but conditions that increase autolysis notably reduce the survival of internalized bacteria. Our assays suggest that the lactobacillus entry process requires actin microtubules and possibly depends upon mechanisms independent from clathrin, caveolin and dynamin canonical endocytic processes, as those described in pathogens and non-pathogenic commensals.

Obviously, BL23 and LGG managed to survive in the cytoplasm of epithelial cells because they reached a good balance between internalization and the escape from cellular (auto)lysis. Finally, this research demonstrated the conditions for spontaneous internalization, intracellular survival and transcytosis, which opens the landscape to future work on the interaction of mutualistic bacteria with the host.

## Figures and Tables

**Table 1:** Transcytosis determined in transwells. This table shows colony forming units found in the basolateral compartment after incubation of Caco-2 monolayers where BL23 and LGG at MOI 10^3^ had been inoculated at the apical compartment and incubated for the indicated time with or without 80 µg/mL Dynasore, as well as Lucifer Yellow (50 μmol/L) to control paracellular diffusion. TEER values shown are the mean determined the day of the experiment for each insert. There were no significant differences between the means (*t* test).

	Mean Values at 2.5 h				Mean Values at 5 h			
	CFU/mL	*Sdev* (*CFU*)	% LY	TEER	CFU/mL	*Sdev* (*CFU*)	%LY	TEER
**CTRL**	-	-	1.01	229.25	-	-	1.13	219.00
**CTRL + Dyn**	-	-	0.43	214.50	-	-	1.40	201.50
**BL23**	70,355.00	75,797.59	0.09	230.00	43,720.00	42,619.50	0.65	222.25
**BL23 + Dyn**	61,050.00	58,889.13	0.42	226.25	44,025.00	29,161.32	1.49	226.00
**LGG**	16,355.00	21,959.19	0.03	237.00	7020.00	3838.78	0.56	228.50
**LGG + Dyn**	31,100.00	22,343.83	0.60	244.25	13,005.00	13,527.80	1.98	226.00

## Data Availability

Not applicable.

## Supplementary Materials

The following supporting information can be downloaded at https://www.mdpi.com/article/10.3390/microorganisms10061142/s1; Figure S1—Effect of different treatments on the internalization of BL23[pT1-GR::p127] in Caco-2 and T84 determined by flow cytometry; Figure S2—Spontaneous internalization (given as CFU/mL) of BL23 and LGG determined by gentamicin protection assays after the treatment; Figure S3—Effect of Caco-2 and T84 treatments with Cytochalasin D (1 and 5 μg/mL), EIPA (50 μM and 25 μM), Rapamycin (10 and 20 ng/mL) on the internalization of BL23[pT1-GR::p127] in Caco-2 and T84; Figure S4—Confocal microscopy photographs showing internalization by T84 of non-viable BL 23 [pT1-GR::p127]; Figure S5—Ability of BL23 and LGG to form Biofilm determined by the plate assay with crystal violet; Table S1—Internalization of BL23 and LGG by IEC is not associated to cell death; Video S1: Confocal screen capture scanning T84 preparation incubated with BL23 expressing GFP.

## References

[1] Hooper L.V., Gordon J.I. (2001). Commensal Host-Bacterial Relationships in the Gut. Science.

[2] do Carmo F.L.R., Rabah H., De Oliveira Carvalho R.D., Gaucher F., Cordeiro B.F., da Silva S.H., Le Loir Y., Azevedo V., Jan G. (2018). Extractable Bacterial Surface Proteins in Probiotic–Host Interaction. Front. Microbiol..

[3] Ruiz L., Hevia A., Bernardo D., Margolles A., Sánchez B. (2014). Extracellular molecular effectors mediating probiotic attributes. FEMS Microbiol. Lett..

[4] Wu L.-L., Peng W.-H., Kuo W.-T., Huang C.-Y., Ni Y.-H., Lu K.-S., Turner J.R., Yu L.C.H. (2014). Commensal Bacterial Endocytosis in Epithelial Cells Is Dependent on Myosin Light Chain Kinase–Activated Brush Border Fanning by Interferon-γ. Am. J. Pathol..

[5] Yu L.C.-H. (2015). Commensal bacterial internalization by epithelial cells: An alternative portal for gut leakiness. Tissue Barriers.

[6] Ladinsky M.S., Araujo L.P., Zhang X., Veltri J., Galan-Diez M., Soualhi S., Lee C., Irie K., Pinker E.Y., Narushima S. (2019). Endocytosis of commensal antigens by intestinal epithelial cells regulates mucosal T cell homeostasis. Science.

[7] Bouchard D.S., Rault L., Berkova N., Le Loir Y., Even S. (2013). Inhibition of *Staphylococcus aureus* invasion into bovine mammary epithelial cells by contact with live *Lactobacillus casei*. Appl. Environ. Microbiol..

[8] Christophe M., Kuczkowska K., Langella P., Eijsink V.G.H., Mathiesen G., Chatel J.-M. (2015). Surface display of an anti-DEC-205 single chain Fv fragment in Lactobacillus plantarum increases internalization and plasmid transfer to dendritic cells in vitro and in vivo. Microb. Cell. Factories.

[9] Souza R.F.S., Jardin J., Cauty C., Rault L., Bouchard D.S., Bermúdez-Humarán L.G., Langella P., Monedero V., Seyffert N., Azevedo V. (2017). Contribution of sortase SrtA2 to Lactobacillus casei BL23 inhibition of *Staphylococcus aureus* internalization into bovine mammary epithelial cells. PLoS ONE.

[10] Guha D., Mukherjee R., Aich P. (2021). Effects of two potential probiotic Lactobacillus bacteria on adipogenesis in vitro. Life Sci..

[11] Machado F.S., Rodriguez N.E., Adesse D., Garzoni L.R., Esper L., Lisanti M.P., Burk R.D., Albanese C., Van Doorslaer K., Weiss L.M. (2012). Recent developments in the interactions between caveolin and pathogens. Adv. Exp. Med. Biol..

[12] Kline K.A., Fälker S., Dahlberg S., Normark S., Henriques-Normark B. (2009). Bacterial Adhesins in Host-Microbe Interactions. Cell Host Microbe.

[13] Ribet D., Cossart P. (2015). How bacterial pathogens colonize their hosts and invade deeper tissues. Microbes Infect..

[14] Kassa E.G., Zlotkin-Rivkin E., Friedman G., Ramachandran R.P., Melamed-Book N., Weiss A.M., Belenky M., Reichmann D., Breuer W., Pal R.R. (2019). Enteropathogenic *Escherichia coli* remodels host endosomes to promote endocytic turnover and breakdown of surface polarity. PLoS Pathog..

[15] Agerer F., Lux S., Michel A., Rohde M., Ohlsen K., Hauck C.R. (2005). Cellular invasion by *Staphylococcus aureus* reveals a functional link between focal adhesion kinase and cortactin in integrin-mediated internalisation. J. Cell Sci..

[16] Kankainen M., Paulin L., Tynkkynen S., von Ossowski I., Reunanen J., Partanen P., Satokari R., Vesterlund S., Hendrickx A.P.A., Lebeer S. (2009). Comparative genomic analysis of *Lactobacillus rhamnosus* GG reveals pili containing a human- mucus binding protein. Proc. Natl. Acad. Sci. USA.

[17] Tytgat H.L.P., van Teijlingen N.H., Sullan R.M.A., Douillard F.P., Rasinkangas P., Messing M., Reunanen J., Satokari R., Vanderleyden J., Dufrêne Y.F. (2016). Probiotic Gut Microbiota Isolate Interacts with Dendritic Cells via Glycosylated Heterotrimeric Pili. PLoS ONE.

[18] Konstantinov S.R., Smidt H., de Vos W.M., Bruijns S.C., Singh S.K., Valence F., Molle D., Lortal S., Altermann E., Klaenhammer T.R. (2008). S layer protein A of Lactobacillus acidophilus NCFM regulates immature dendritic cell and T cell functions. Proc. Natl. Acad. Sci. USA.

[19] Sillanpaa J., Martinez B., Antikainen J., Toba T., Kalkkinen N., Tankka S., Lounatmaa K., Keranen J., Hook M., Westerlund-Wikstrom B. (2000). Characterization of the Collagen-Binding S-Layer Protein CbsA of *Lactobacillus crispatus*. J. Bacteriol..

[20] Wang L., Si W., Xue H., Zhao X. (2017). A fibronectin-binding protein (FbpA) of *Weissella cibaria* inhibits colonization and infection of *Staphylococcus aureus* in mammary glands. Cell. Microbiol..

[21] Botha M., Botes M., Loos B., Smith C., Dicks L.M.T. (2012). *Lactobacillus equigenerosi* strain Le1 invades equine epithelial cells. Appl. Environ. Microbiol..

[22] Leccese Terraf M.C., Juarez Tomás M.S., Rault L., Le Loir Y., Even S., Nader-Macías M.E.F. (2017). In vitro effect of vaginal lactobacilli on the growth and adhesion abilities of uropathogenic *Escherichia coli*. Arch. Microbiol..

[23] Tian T., Zhu Y.-L., Zhou Y.-Y., Liang G.-F., Wang Y.-Y., Hu F.-H., Xiao Z.-D. (2014). Exosome Uptake through Clathrin-mediated Endocytosis and Macropinocytosis and Mediating miR-21 Delivery. J. Biol. Chem..

[24] Almeida J.F., Mariat D., Azevedo V., Miyoshi A., de Moreno de LeBlanc A., Del Carmen S., Martin R., Langella P., LeBlanc J.-G., Chatel J.-M. (2014). Correlation between fibronectin binding protein a expression level at the surface of recombinant *Lactococcus lactis* and plasmid transfer in vitro and in vivo. BMC Microbiol..

[25] Guimarães V.D., Gabriel J.E., Lefèvre F., Cabanes D., Gruss A., Cossart P., Azevedo V., Langella P. (2005). Internalin-expressing *Lactococcus lactis* is able to invade small intestine of guinea pigs and deliver DNA into mammalian epithelial cells. Microbes Infect..

[26] Innocentin S., Guimarães V., Miyoshi A., Azevedo V., Langella P., Chatel J.-M., Lefèvre F. (2009). *Lactococcus lactis* Expressing either *Staphylococcus aureus* Fibronectin-Binding Protein A or *Listeria monocytogenes* Internalin a Can Efficiently Internalize and Deliver DNA in Human Epithelial Cells. Appl. Environ. Microbiol..

[27] Pontes D., Innocentin S., del Carmen S., Almeida J.F., LeBlanc J.-G., de Moreno de LeBlanc A., Blugeon S., Cherbuy C., Lefèvre F., Azevedo V. (2012). Production of Fibronectin Binding Protein A at the Surface of *Lactococcus lactis* Increases Plasmid Transfer In Vitro and In Vivo. PLoS ONE.

[28] Mathipa M.G., Thantsha M.S., Bhunia A.K. (2019). Lactobacillus casei expressing Internalins A and B reduces *Listeria monocytogenes* interaction with Caco-2 cells in vitro. Microb. Biotechnol..

[29] Zocco M.A., Dal Verme L.Z., Cremonini F., Piscaglia A.C., Nista E.C., Candelli M., Novi M., Rigante D., Cazzato I.A., Ojetti V. (2006). Efficacy of Lactobacillus GG in maintaining remission of ulcerative colitis. Aliment. Pharm. Ther..

[30] Szajewska H., Wanke M., Patro B. (2011). Meta-analysis: The effects of *Lactobacillus rhamnosus* GG supplementation for the prevention of healthcare-associated diarrhoea in children. Aliment. Pharm. Ther..

[31] McFarland V.L. (2007). Meta-analysis of probiotics for the prevention of traveler’s diarrhea. Travel Med. Infect. Dis..

[32] Dietrich C., Kottmann T., Alavi M. (2014). Commercially available probiotic drinks containing *Lactobacillus casei* DN-114001 reduce antibiotic-associated diarrhea. World J. Gastroenterol..

[33] Lee B., Yin X., Griffey S.M., Marco M.L. (2015). Attenuation of colitis by *Lactobacillus casei* BL23 is dependent on the dairy delivery matrix. Appl. Environ. Microbiol..

[34] Auclair J., Frappier M., Millette M. (2015). *Lactobacillus acidophilus* CL1285, *Lactobacillus casei* LBC80R, and *Lactobacillus rhamnosus* CLR2 (Bio-K+): Characterization, Manufacture, Mechanisms of Action, and Quality Control of a Specific Probiotic Combination for Primary Prevention of *Clostridium difficile* Infection. Clin. Infect. Dis..

[35] Hirano J., Yoshida T., Sugiyama T., Koide N., Mori I., Yokochi T. (2003). The effect of *Lactobacillus rhamnosus* on enterohemorrhagic *Escherichia coli* infection of human intestinal cells in vitro. Microbiol. Immunol..

[36] Vargas García C.E., Petrova M., Claes I.J.J., De Boeck I., Verhoeven T.L.A., Dilissen E., von Ossowski I., Palva A., Bullens D.M., Vanderleyden J. (2015). Piliation of *Lactobacillus rhamnosus* GG Promotes Adhesion, Phagocytosis, and Cytokine Modulation in Macrophages. Appl. Environ. Microbiol..

[37] Lebeer S., Claes I., Tytgat H.L.P., Verhoeven T.L.A., Marien E., von Ossowski I., Reunanen J., Palva A., Vos W.M.d., Keersmaecker S.C.J.D. (2012). Functional analysis of *Lactobacillus rhamnosus* GG pili in relation to adhesion and immunomodulatory interactions with intestinal epithelial cells. Appl. Environ. Microbiol..

[38] Tripathi P., Beaussart A., Alsteens D., Dupres V., Claes I., von Ossowski I., de Vos W.M., Palva A., Lebeer S., Vanderleyden J. (2013). Adhesion and Nanomechanics of Pili from the Probiotic *Lactobacillus rhamnosus* GG. ACS Nano.

[39] Coll-Marqués J.M., Bäuerl C., Zúñiga M., Pérez-Martínez G. (2020). Differences in the expression of cell envelope proteinases (CEP) in two *Lactobacillus paracasei* probiotic strains. FEMS Microbiol. Lett..

[40] Bäuerl C., Abitayeva G., Sosa-Carrillo S., Mencher-Beltrán A., Navarro-Lleó N., Coll-Marqués J.M., Zúñiga-Cabrera M., Shaikhin S., Pérez-Martinez G. (2019). P40 and P75 Are Singular Functional Muramidases Present in the *Lactobacillus casei*/*paracasei*/*rhamnosus* Taxon. Front. Microbiol..

[41] Clark E., Hoare C., Tanianis-Hughes J., Carlson G.L., Warhurst G. (2005). Interferon γ Induces Translocation of Commensal *Escherichia coli* Across Gut Epithelial Cells via a Lipid Raft—Mediated Process. Gastroenterology.

[42] Kalischuk L.D., Inglis G.D., Buret A.G. (2009). *Campylobacter jejuni* induces transcellular translocation of commensal bacteria via lipid rafts. Gut Pathog..

[43] Srinivasan B., Kolli A.R., Esch M.B., Abaci H.E., Shuler M.L., Hickman J.J. (2015). TEER measurement techniques for in vitro barrier model systems. J. Lab. Autom..

[44] Ferruzza S., Rossi C., Scarino M.L., Sambuy Y. (2012). A protocol for in situ enzyme assays to assess the differentiation of human intestinal Caco-2 cells. Toxicol. In Vitro.

[45] Bäuerl C., Coll-Marqués J.M., Tarazona-González C., Pérez-Martínez G. (2020). *Lactobacillus casei* extracellular vesicles stimulate EGFR pathway likely due to the presence of proteins P40 and P75 bound to their surface. Sci. Rep..

[46] Fan Y., Pedersen O. (2021). Gut microbiota in human metabolic health and disease. Nat. Rev. Microbiol..

[47] Ikari A., Takiguchi A., Atomi K., Sugatani J. (2011). Epidermal growth factor increases clathrin-dependent endocytosis and degradation of claudin-2 protein in MDCK II cells. J. Cell. Physiol..

[48] Yoshida S., Pacitto R., Inoki K., Swanson J. (2018). Macropinocytosis, mTORC1 and cellular growth control. Cell. Mol. Life Sci..

[49] Yan F., Cao H., Cover T.L., Whitehead R., Washington M.K., Polk D.B. (2007). Soluble Proteins Produced by Probiotic Bacteria Regulate Intestinal Epithelial Cell Survival and Growth. Gastroenterology.

[50] Lu R., Shang M., Zhang Y.G., Jiao Y., Xia Y., Garrett S., Bakke D., Bäuerl C., Martinez G.P., Kim C.H. (2020). Lactic Acid Bacteria Isolated from Korean Kimchi Activate the Vitamin D Receptor-autophagy Signaling Pathways. Inflamm. Bowel Dis..

[51] Yeruva S., Ramadori G., Raddatz D. (2008). NF-κB-dependent synergistic regulation of CXCL10 gene expression by IL-1β and IFN-γ in human intestinal epithelial cell lines. Int. J. Colorectal Dis..

[52] Paulus G.L.C., Xavier R.J. (2015). Autophagy and checkpoints for intracellular pathogen defense. Curr. Opin. Gastroenterol..

[53] Smyth D., McKay C.M., Gulbransen B.D., Phan V.C., Wang A., McKay D.M. (2012). Interferon-gamma signals via an ERK1/2-ARF6 pathway to promote bacterial internalization by gut epithelia. Cell. Microbiol..

[54] Lamaze C., Fujimoto L.M., Yin H.L., Schmid S.L. (1997). The Actin Cytoskeleton Is Required for Receptor-mediated Endocytosis in Mammalian Cells. J. Biol. Chem..

[55] Gottlieb T.A., Ivanov I.E., Adesnik M., Sabatini D.D. (1993). Actin microfilaments play a critical role in endocytosis at the apical but not the basolateral surface of polarized epithelial cells. J. Cell. Biol..

[56] Olazabal I.M., Caron E., May R.C., Schilling K., Knecht D.A., Machesky L.M. (2002). Rho-Kinase and Myosin-II Control Phagocytic Cup Formation during CR, but Not FcγR, Phagocytosis. Curr. Biol..

[57] Preta G., Cronin J.G., Sheldon I.M. (2015). Dynasore-not just a dynamin inhibitor. Cell. Commun. Signal.

[58] Loh L.N., Gao G., Tuomanen E.I. (2017). Dissecting Bacterial Cell Wall Entry and Signaling in Eukaryotic Cells: An Actin-Dependent Pathway Parallels Platelet-Activating Factor Receptor-Mediated Endocytosis. mBio.

