# Supplementary material for: Factors Affecting Spontaneous Endocytosis and Survival of Probiotic Lactobacilli in Human Intestinal Epithelial Cells
Source: Microorganisms. 2022 May 31;10(6):1142. doi: 10.3390/microorganisms10061142 (PMC9230732; doi:10.3390/microorganisms10061142)
Supplement: Supplementary file 1 [file microorganisms-10-01142-s001.zip › microorganisms-1725115-supplementary-Figures-Tables.pdf]

Factors Affecting Spontaneous Endocytosis and Survival of Probiotic Lactobacilli in Human Intestinal Epithelial Cells

Diana Aurora Ramirez-Sánchez[1§], Noemi Navarro-Lleó[2§], Chistine Bäuerl[3], Samuel Campista-León[1], José María Coll-Marqués[3] and Gaspar Pérez Martínez[3*]

[1] Facultad de Biología, Universidad Autónoma de Sinaloa, Culiacán Rosales, Sinaloa, México; DRS, diana-ramirez-sanchez@hotmail.com, SCL, calsa68@hotmail.com

[2] Department of Microbiology and Ecology, Faculty of Medicine, University of Valencia, Spain; NNL, noemi.navarro@uv.es

[3] Laboratory of Lactic acid and Probiotics, Department of Biotechnology, Instituto de Agroquímica y Tecnología de Alimentos, Consejo Superior de Investigaciones Científicas (CSIC) (Spanish National Research Council), Valencia, Spain; CB, cbauerl@iata.csic.es; JMCM, jcoll@iata.csic.es; GPM, gaspar.perez@iata.csic.es

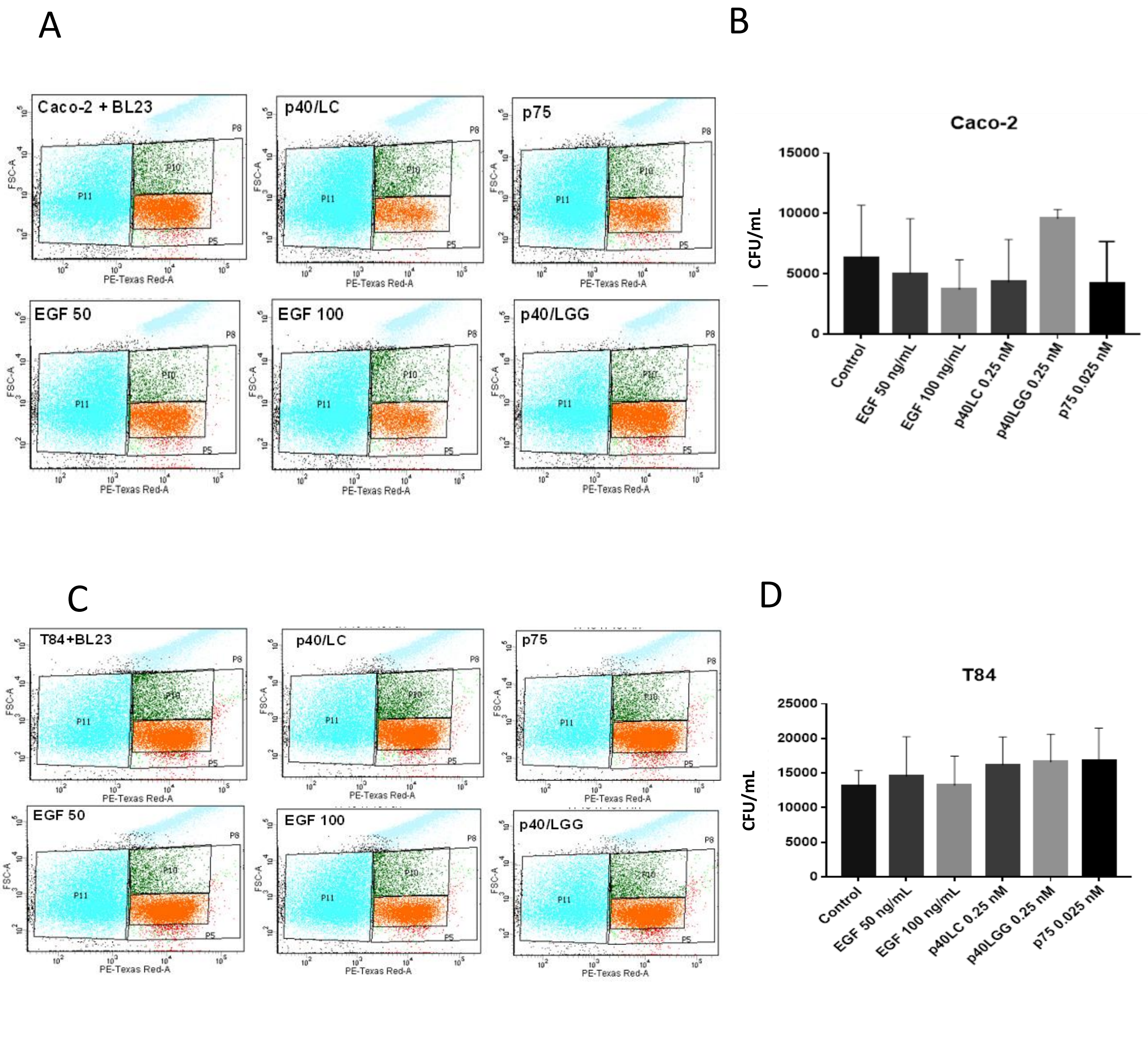

Figure S1.- Effect of different treatments on the internalization of BL23[pT1-GR::p127] in Caco-2 and T84. Treatments included: BL23 [pT1-GR::p127] control, *L. paracasei* BL23 P40 and P75, *L. rhamnosus* GG P40 (P40LGG)(0.25 nM/mL) and EGF (50 and 100 ng/mL). (A) Flow cytometry scatter plot of representative experiments of Caco-2; (B) Data from Caco-2 aassays presented as the means of the number of fluorescent events of the P8 region including standard deviation barr; (C) and (D) the same as (A and B) but with T84. The experiments were performed with two replicates. Group differences determined by the Bonferroni test.

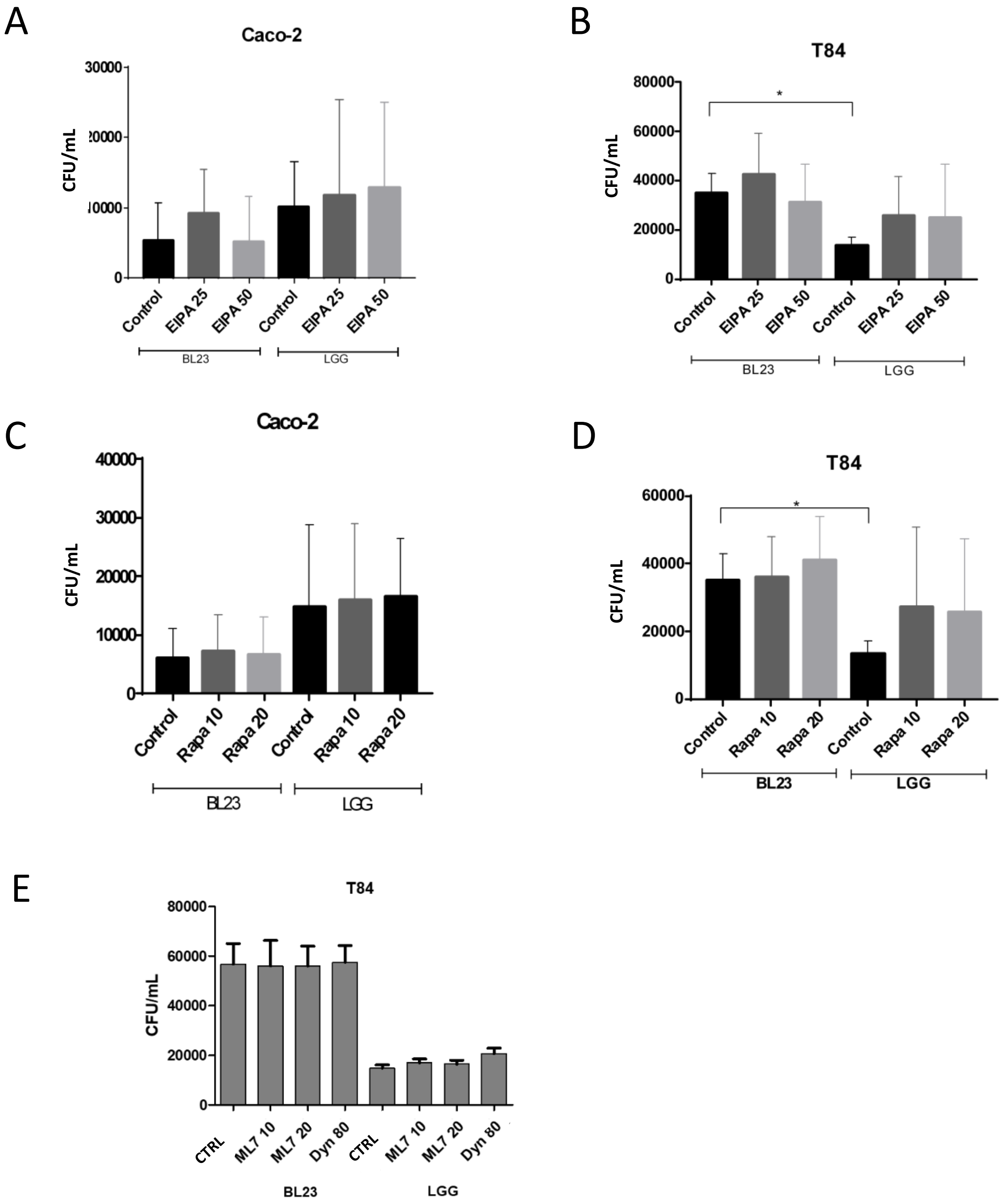

Figure S2.- Spontaneous internalization (given as CFU/mL) of BL23 and LGG determined by gentamycin protection assays after the treatment of : (A) Caco-2 with EIPA (50 µM and 25 µM); (B) T84 with EIPA (50 µM and 25 µM); (C) Caco-2 with Rapamycin (10 and 20 ng/mL); (D) T84 with Rapamycin (10 and 20 ng/mL); (E) T84 with ML7 (10 and 20 µg/mL) and Dynamin (80 µM). (results from four separate assays, with three internal replicates per condition). Differences between groups compared with Tamhane's test. *P < 0.001.

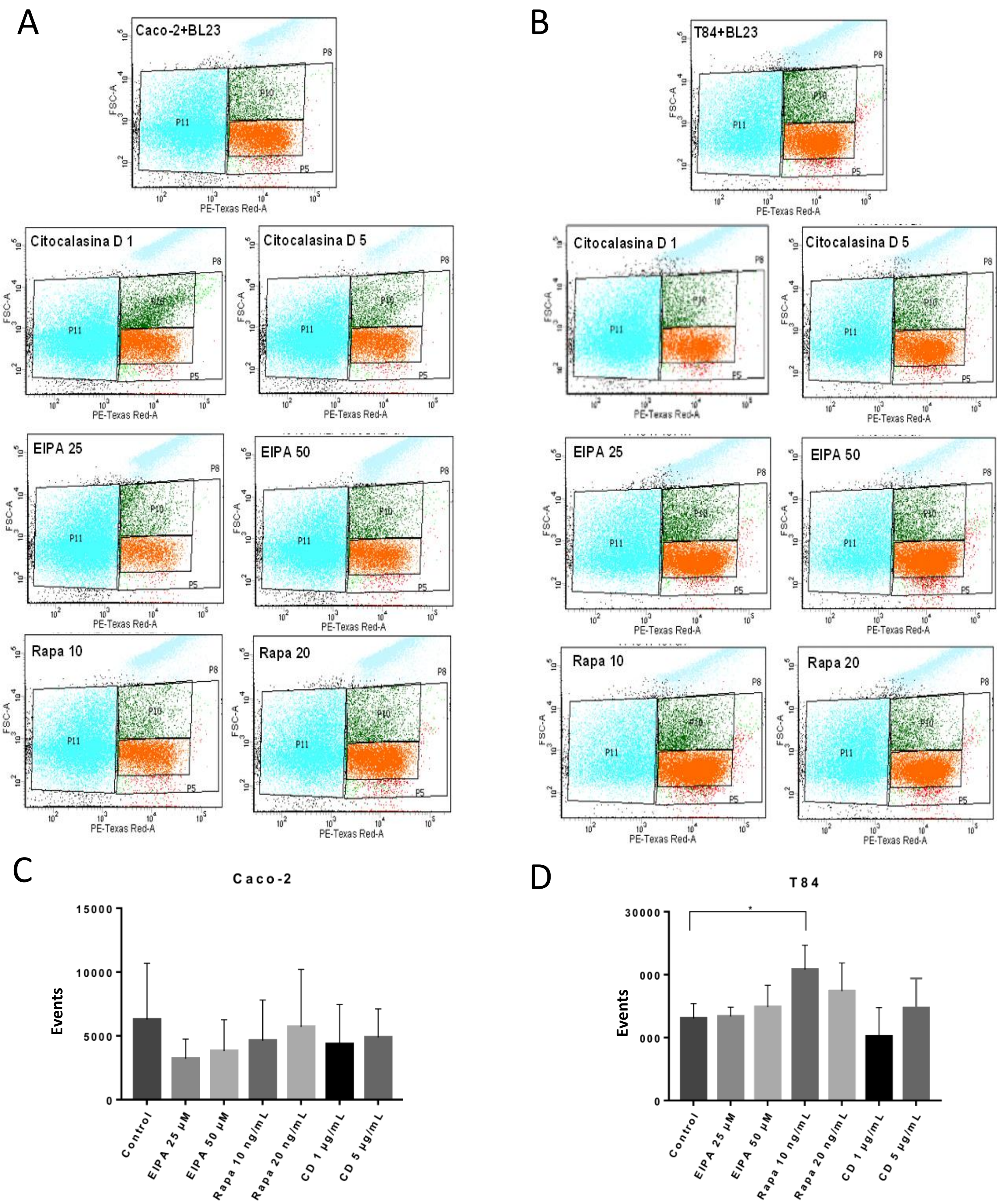

Figure S3.- Effect of Caco-2 and T84 treatments with Cytochalasin D (1 and 5 µg/mL), EIPA (50 µM and 25 µM), Rapamycin (10 and 20 ng/mL) on the internalization of BL23[pT1-GR::p127] in Caco-2 and T84. (A) Flow cytometry scatter plot of a representative experiment of Caco-2; (B) scatter plot of a representative experiment of T84; (C) Data from Caco-2 and (D) T84 assays presented as the means of the number of fluorescent events of the P8 region including standard deviation barr. The experiments were performed with two replicates. Group differences determined by the Bonferroni test (*P<0.05).

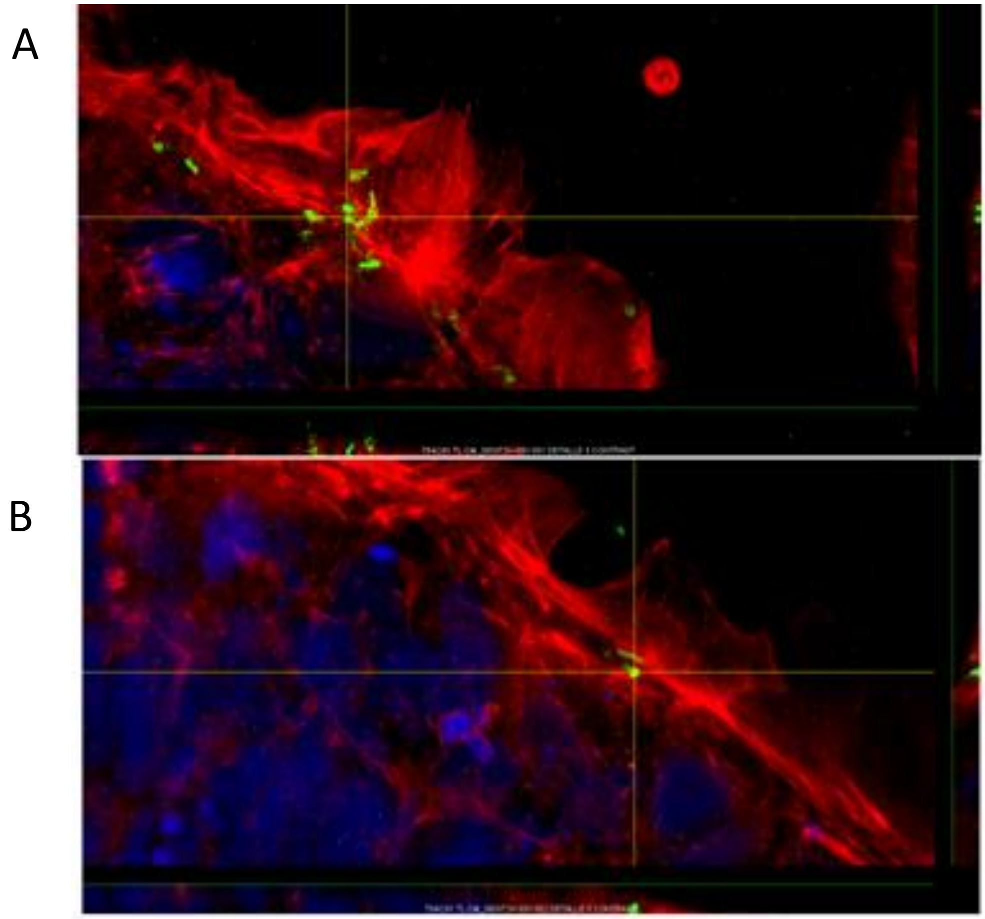

Figure S4.- Confocal microscopy photographs showing internalization by T84 of non-viable BL 23 [pT1-GR::p127] treated with chloramphenicol (25 µg/mL) and gentamycin (300 µg/mL). Fluorophores legend: DAPI in blue, GFP in green, Actin in red. Objective used: 60x

---

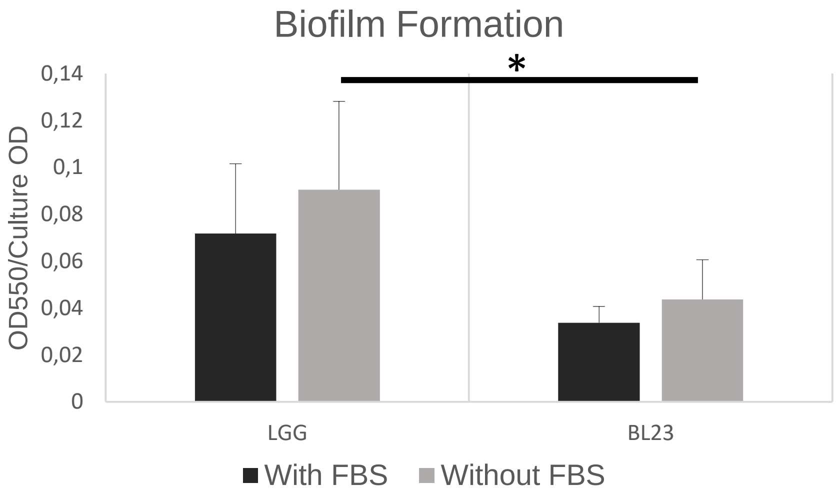

Figure S5.- Ability to form Biofilm determined by the plate assay with crystal violet. Graph representing colour intensity attached to the ELISA plate when assaying *L. paracasei* BL23 and *L. rhamnosus* GG on DMEM supplemented with faetal bovine serum (FBS) or not. Group differences determined by the t test (*P<0.05).

Table S1- Internalization of BL23 and LGG by IEC is not associated to cell death. This table shows the average of at least three fields per sample after different times of incubation with bacteria, where the following were scored: DAPI-stained nuclei, propidium iodide labelled nuclei, BL23 [pT1-GR::p127] (GFP) in groups or individual bacteria.

Sample	Incubation time	Average viable nuclei (DAPI) per field	Average PI stained nuclei	Average BL23 [pT1-GR::p127] clusters	Average individual BL23 [pT1-GR::p127] cells	Association rate of BL23 [pT1-GR::p127] with PI nuclei
Caco-2	4	157	0	0	0	0.0
Caco-2 + BL23	4	113	0	3	5	0.0
Caco-2	24	73	0	0	0	0.0
Caco-2 + BL23	24	72	0	5	9	0.0
Caco-2	48	240	2	0	0	0.0
Caco-2 + BL23	48	128	0	3	8	0.0
T84	4	100	0	0	0	0.0
T84+ BL23	4	72	0	13	43	0.0
T84	24	140	1	0	0	0.0
T84+ BL23	24	116	1	12	39	0.3
T84	48	143	1	0	0	0.0
T84+ BL23	48	83	1	7	24	0.0
